# Will the Foot Strike Pattern Change at Different Running Speeds with or without Wearing Shoes?

**DOI:** 10.3390/ijerph17176044

**Published:** 2020-08-20

**Authors:** Ying-Jen Lai, Willy Chou, I-Hua Chu, Yu-Lin Wang, Yi-Jing Lin, Shihfan Jack Tu, Lan-Yuen Guo

**Affiliations:** 1 Department of Sports Medicine, College of Medicine, Kaohsiung Medical University, Kaohsiung 807, Taiwan; zan306704@yahoo.com.tw (Y.-J.L.); ihchu@kmu.edu.tw (I.-H.C.); toto8052t2010@hotmail.com (Y.-J.L.); jackshtu@gmail.com (S.J.T.); 2 Department of Physical Medicine and Rehabilitation, Chi Mei Medical Center, Tainan 710, Taiwan; ufan0101@ms22.hinet.net (W.C.); d8101080@gmail.com (Y.-L.W.); 3 Department of Physical Medicine and Rehabilitation, Chung Shan Medical University, Taichung 402, Taiwan; 4 Program in Biomedical Engineering, College of Medicine, Kaohsiung Medical University, Kaohsiung 807, Taiwan; 5 Department of Medical Research, Kaohsiung Medical University Hospital, Kaohsiung 807, Taiwan

**Keywords:** barefoot running, foot strike pattern, forefoot strike, midfoot strike, rearfoot strike

## Abstract

Runners strike their feet with three different patterns during running: forefoot, midfoot, and rearfoot. This study aimed to investigate whether runners maintain consistent patterns while running speed and foot condition change. The foot strike patterns of runners when running on a treadmill at paces ranging from slow to fast were recorded from twenty healthy male regular runners, with and without shoes, in random order. A high-speed camera was used to observe the strike patterns, which were then categorized by an experienced physical therapist. Linear-log and Pearson chi-square analysis with a significance level of α = 0.05 was performed to examine the correlation between foot strike pattern, running speed, and shoe conditions. The results suggest that runners strike with different patterns when running with and without shoes (χ^2^ = 99.07, *p* < 0.01); runners preferred to adopt heel strike regardless of running speeds when running with shoes. While running barefoot, only 23.8% of landing strikes were rearfoot, and the strike pattern distribution did not change significantly with the running speed (χ^2^ = 2.26, *p* = 0.89). In summary, the foot strike preference of runners is correlated with the foot condition (barefoot or shod) rather than running speed. For runners who intend to change their strike patterns for any reason, we recommend that they consider adjusting their footwear, which may naturally help with the foot strike adjustment. Future studies should attempt to use advanced techniques to observe further foot biomechanics in order to discover if changing strike pattern is directly correlated with lower limb injuries.

## 1. Introduction

Running has become one of the most popular aerobic exercises, although many potential injuries related to running have been well studied [1]. A systematic review reported that the incidence of running injuries in long-distance runners varied from 19.4% to 92.4% [1]. Many authors have reported that the most common site of lower extremity running injuries is the knee, and running-related injuries result from a complex of risk factors, such as training errors, inappropriate surface, lower limb biomechanics, footwear, etc. [2,3,4]. It is believed that the most injurious moment of running is when the foot strikes the ground, as the lower limbs need to transmit and absorb energy during the collision. Therefore, reducing loads on feet to prevent running-related injuries has remained an area of research interest for more than 20 years [5,6]. Some believe that newly developed footwear is better for support and absorbing vibration and reaction forces, as the manufacturing techniques have gradually improved in past decades. However, Taunton et al. [3] reported that running-related injury rates have not reduced in comparison with the rates over 20 years reported in an earlier study [7]. Additionally, there is no difference in ground reaction forces (GRFs) between barefoot and shod running [8]; instead, habitually shod runners may strike differently than barefoot runners do [5]. Thus, the landing pattern seems to be more relevant than footwear in terms of reducing ground reaction force during running.

It is therefore necessary to understand how runners’ feet make contact with the ground during the takeoff and stance phases, as the foot is the only body segment that directly supplies force to the ground during running cycles. Foot strike during landing is usually classified into three patterns: rearfoot strike (RFS), midfoot strike (MFS), and forefoot strike (FFS) [9]. Cavanagh and Lafortune [10] demonstrated that the RFS induced maximum ground reaction force while MFS initiated lower ground reaction force during foot landing. Later studies agreed with this statement. Lieberman et al. [5] claimed that landing with the forefoot or midfoot, particularly in those who run barefoot, can achieve an optimal cushioning effect naturally; therefore, this may reduce the risk of lower limb injuries. Shih et al. [8] also reported that the ground reaction force was significantly reduced when landing with the forefoot in comparison with the rearfoot. Moreover, the ground reaction force was particularly high when running barefoot and landing with rearfoot strikes [8]. Given these findings, the foot strike pattern during running should be carefully considered in the prevention of running-related injury.

Running speed is considered to be correlated with foot strike pattern. Hasegawa et al. [9] found that most endurance runners used RFS, while sprinters usually used FFS. They also found a trend towards RFS in faster half-marathon runners. Similarly, middle-distance runners were found to use FFS more often [11]. These results can be explained by these competitive runners using specifically designed shoes to increase running speed, and having matched strike patterns. Other studies demonstrated that runners did not maintain the same strike pattern all the time; runners who mostly used RFS at slower speeds shifted towards a more midfoot or forefoot strike at higher speeds—faster than 18 km/h [12,13]. A recent study reported a foot landing pattern distribution in recreational runners running at different speeds; the rearfoot–midfoot ratio was around 3:1 at lower speed and switched to 1:1 at higher speed [14]. These findings suggest an idea that landing patterns are highly related to running speed; running with non-RFS seems to be a way to enable runners either to cope with the collision force related to faster running or to enhance running economics [13,15].

To summarize, previous studies have provided adequate evidence of running biomechanics, such as GRF, and strike kinematics in relation to barefoot or shod conditions; however, few studies have discussed how recreational runners strike to cope with speed changes. This study aimed to investigate whether runners maintained consistent patterns while running speed and foot condition changed. Runners were hypothesized to switch from RFS to MFS or FFS when running at a higher speed, and runners may have different landing pattern profile between running barefoot and with shoes. 

## 2. Materials and Methods 

### 2.1. Participants

Twenty healthy male regular runners aged 20 to 40 years were recruited, with a mean height of 172.2 ± 4.2 cm and body mass of 68.5 ± 6.3 kg (BMI 23.1 ± 1.5). All participants were regular recreational runners who ran at least three times per week, and in at least 20 min per session, reached the targeted heart rate. Participants who had a smoking history and musculoskeletal disorders within 6 months prior to the investigation were excluded. A Physical Activity Readiness Questionnaire (PAR-Q) was used to evaluate possible risks of undertaking this running trial for the participants to ensure their safety. The study procedures were approved by the Institutional Review Board of Kaohsiung Medical University Hospital with ethical approval number KMUH-IRB-20130302, and all participants provided written consent before the study. Professional physical therapists and coaches monitored the whole experimental procedure. The experiment was terminated immediately if any incident occurred or discomfort was reported to ensure the safety of the participants. 

Participants were required to run barefoot and with shoes in random order. A 20-min break between two runs was given to minimize fatigue effects. Participants ran on a treadmill (Mercury, h/p/cosmos, Nussdorf-Traunstein, Germany) to control running speeds. This treadmill speed was calibrated according to the manual guideline of the manufacturer before the experimental setup with speed accuracy of ±5% [16,17]. A high-speed camera (HERO3 + Black Edition, GoPro Inc., San Mateo, CA, USA) with a slow-motion module (WVG/240) was used to record foot landing patterns of participants during the running sessions. The camera was placed parallel to the sagittal plane of the runners and focused on a point for a clear visual field of the initial foot contact on the ground [9,18].

### 2.2. Experiment Protocols 

All participants were asked to wear the same sports shoes according to their specific foot size. The sports shoes were similar to regular ones with heel wedges. In each running session, participants initially walked at minimal speed and accelerated by 0.5 km per hour every 15 s. The foot strike observation was performed at four speeds: initial, slow, fast, and ultimate. The initial running speed was recorded when the participant had to switch from walking to running. The test was completed when the participant could no longer increase their running speed, and final speed was defined as the ultimate speed. As runners may have different initial and ultimate speeds, a linear scale was used for normalization. Two intermediate speeds (slow and fast) were defined as one-third of the interval from each end between the initial and ultimate speed. There is some support in the literature for using this method. First, the speed dependence variables in running might have a linear relationship with the energy expenditure variable. In 2000, Greiwe et al. found that oxygen consumption increased linearly with running speed [19]. Second, human locomotion may exist some distinct sets of locomotor modules which control the speed; that is, the same locomotor mode may be the same, even at different speeds [20,21]. This method is based on the concept of normalizing the speed with the ultimate speed, which is one of the parameters for the best performance [22]. The same test with different shoe conditions was performed twice and a 20-min break was given to avoid fatigue effects. The average ultimate speed was 15.24 ± 1.68 km hr^−1^ (i.e., 4.23 ± 0.47 m·s^−1^) and 14.74 ± 1.38 km hr^−1^ (i.e., 4.09 ± 0.13 m·s^−1^) for running with shoe or barefoot, respectively. Initial running speed was around 1.60 m·s^−1^, the slow running speed was around 2.43–2.48 m·s^−1^, and fast running speed was around 3.26–3.35 m·s^−1^, which were similar to the classification in the literature [21]. In 2016, Yokoyama extracted three locomotor modules during running and named them slow running (2.7–2.9 m·s^−1^), moderate running (3.5–3.7 m·s^−1^) and fast running (4.4–4.5 m·s^−1^) for runners [21]. Meanwhile, for non-runners, three locomotor modules were extracted as slow running (2.4–2.7 m·s^−1^), moderate running (3.0–3.3 m·s^−1^) and fast running (3.8–4.0 m·s^−1^) [21].

### 2.3. Data Analysis

**Figure 1:**
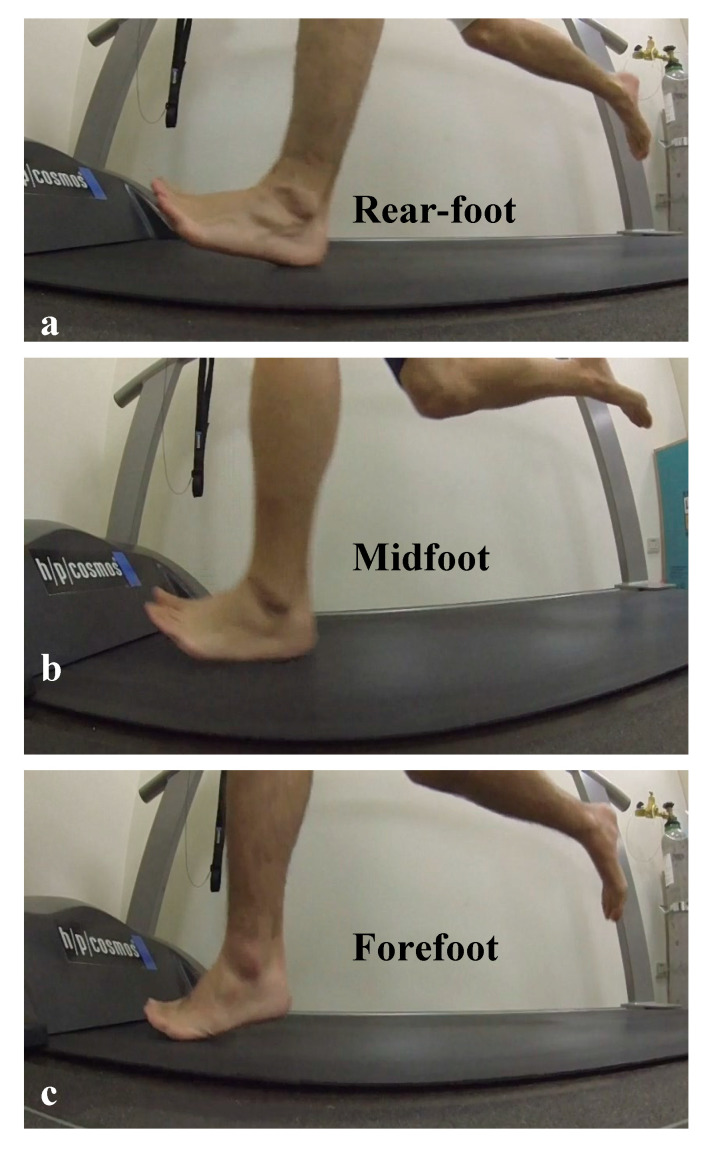
Three common types of landing pattern while running. (**a**): rearfoot strike (RFS), (**b**): midfoot strike (MFS), (**c**):forefoot strike (FFS).

Experimental videos were edited with Power Director (ver. 9, CyberLink Corp, New Taipei, Taiwan) to extract strike pattern information. Clips containing the last 10 s of each running speed were extracted from the video, and types of landing patterns were determined by an experienced physical therapist. The patterns were: rearfoot strike (RFS), in which the heel lands first (Figure 1a); midfoot strike (MFS), in which the heel and ball of the foot land simultaneously (Figure 1b); and forefoot strike (FFS), in which the ball of the foot lands first (Figure 1c) [9,18,23,24]. The strike type with the highest percentage of appearance during the period was considered the preferred strike type for the participant for this particular condition. This strike analysis using 2D imaging was reported with high reliability [23].

The observation results were converted into a three-dimensional contingency table, and log-linear model analysis was applied to explore if the strike pattern preferences of the participants were associated with the shoe condition and running speeds. Partial effects were determined after Pearson chi-square detected the K-way significance to explore the main factor and the interactions. The multivariate linear analysis was performed on SPSS Statistics for Windows, version 19 (IBM Corp., Armonk, NY, USA). The level of significance was set at α = 0.05.

## 3. Results

**Figure 2:**
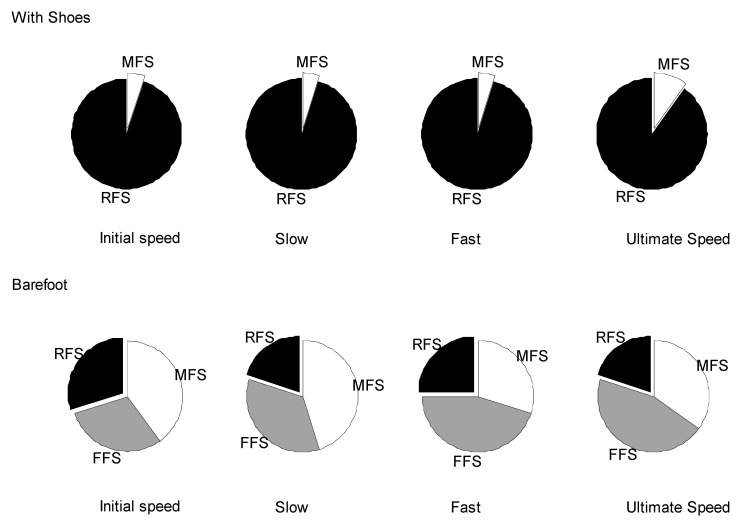
Frequency distribution of three landing patterns when running with or without shoes at four different speeds (FFS: forefoot strike, MFS: midfoot strike, RFS: rearfoot strike).

Figure 2 illustrates the results of the strike pattern observations in which the participants were instructed to run in two conditions at four speeds. The RFS was the most commonly performed landing pattern when running with shoes. Ninety-five percent of runners used RFS at initial, slow, and fast speed, which was only reduced by 5% at ultimate speed. Strike pattern was distributed similarly between the three types when running barefoot. FFS and MFS were similarly preferred (30–45%) followed by RFS, which involved 20 to 30% of participants at all speeds.

Table 1 presents details of strike observation at four running speeds with and without shoes. The log-linear analysis (Table 2) showed no significant correlation between strike pattern preference and running speed changes (χ^2^ = 2.26, *p* = 0.89). Conversely, strike pattern was significantly correlated with shoe conditions (χ^2^ = 99.07, *p* < 0.01). No interaction between shoe condition and running speed was detected in three-way effect analysis (χ^2^ = 0.44, *p* = 0.99).

## 4. Discussion

The aim of the present study was to investigate if runners would maintain the same strike preference when running from slow to fast pace, and additionally if running barefoot or with shoes would change their strike pattern profile. Previous studies have shown that strike pattern is the main factor affecting GRF on the foot, and professional runners tend to run in a specific pattern as per their running type and speed [9,11]. This concept may be applied to injury prevention in recreational running cohorts. However, reports on how recreational runners strike while running in different conditions and speeds are inadequate. Since running was, and continues to be, a popular recreational exercise, discovering this information could contribute to runner education and injury prevention.

One of the aims of this study was to observe if runners would strike differently when running with or without shoes. The results showed that even though 93.8% of runners used RFS when running with shoes, most of them switched to non-RFS when running barefoot (76.3%). Although we did not investigate whether the runners in the present study were habitually barefoot or wearing shoes during their daily lives, the results support the findings of a previous study [5] that reported that habitual barefoot runners tend to avoid RFS in order to cope with the impact from the ground. Even though RFS landing can produce higher GRF and a greater level of impact, irritating the foot [5,10], it can be assumed that the shoes provided in the present study were able to absorb the impacts of GRF to improve foot comfort during running. Lieberman et al. [5] reported that shod RFS runners had a similar mean rate of loading (i.e., impact force normalized to time) as barefoot FFS runners, regardless of there being a difference in absolute impact force. This finding may support the hypothesis that most participants were able to maintain RFS at all times when running with shoes due to the cushioning effect of the shoes.

The results indicate that increasing running speed did not change strike patterns during running. When the participant was running barefoot, the percentage of FFS showed a trend towards increasing with increasing running speeds, albeit with insignificance in the linear analysis (χ^2^ = 2.26, *p* = 0.89). However, participants almost ran with RFS at all times when wearing shoes. No interaction between shoe condition and running speeds was found (χ^2^ = 0.44, *p* = 0.99), which indicated that speed did not influence the difference in foot strike patterns between barefoot and shod running. These findings do not entirely corroborate the hypothesis and the literature, which demonstrated that running at faster speeds would lead the runners to use FFS instead of RFS [12,13,14]. We assumed that the participants slightly altered the lower limb kinematics while running on a treadmill with consistently increasing speeds. A previous study demonstrated that when running speed increased, the angles of hip, knee, and ankle joints increased during the swing phase of running in order to lengthen stride distances and reach higher speed [25]. This may be a reason why some participants did not switch their foot strike patterns towards MFS or FFS as we hypothesized.

There were a few limitations to the present study. Firstly, running on a treadmill could have affected the running performance of the runners, as they needed to pay extra attention to maintaining speed and stabilizing the body to adapt to the moving belt [26]. Lack of natural scenes being a visual reference when running on a treadmill can be another factor affecting balance and stability performance [26,27,28,29]. For example, participants usually shorten gait strides and amplify step frequency when walking or running on a treadmill [30,31,32,33]. Thus, the results of the present study need to be treated carefully when applied to road running.

Categorizing strike types by visual observation with a high-speed camera and slow-motion play-back is a time-consuming method, although it is an alternative method to avoid self-reported foot strike pattern identification, which has been reported to be insufficiently accurate [34]. In the present study, the same physical therapist consistently examined all the videos and was blinded to the running conditions in order to ensure reliability and prevent bias. It is a straightforward method to be used in such a controlled situation, although we did not inspect the inter-rater reliability. The current study included 20 young male subjects. Confidence in the conclusions of the study as well as application to different populations may be achieved by increasing the sample size and including participants of different age and gender. Meanwhile, a future study could consider applying foot pressure sensors, underbelt force plates, or two-dimensional cameras, and categorizing strike patterns automatically to improve such concerns. Iso-efficiency speed may be a good concept to consider using the metabolic demand, such as VO_2_max or HRmax, as a normalization method to determine the speed effect [22]. This study focused on the foot strike pattern analysis; thereafter, a 2D video analysis in the sagittal plane was chosen for the overground running measures. Those variables, including contact time, flight time, stride frequency, and stride length, which might provide more insight into running strike analysis, were not collected or analyzed due to equipment limitations or lack of understanding of the reliability of using 2D video analyses [23,35,36]. While running-related overuse injuries have been considered as an important issue, knowledge of the foot strike pattern would appear to be important information for the clinician managing a runner with a running-related overuse injury.

## 5. Conclusions

The present study demonstrated that running with or without shoes seems to be the main factor affecting the preference for foot strike patterns for young male subjects running on a treadmill. Although it is believed that running speed is highly correlated with the foot strike pattern, the results seem to reject this statement. The appearance of non-RFS only increased slightly when participants were running at the ultimate speed. Therefore, shoes should be primarily considered if runners intend to adjust their strike pattern for any reason. Future studies should investigate further biomechanics with advanced techniques, such as full-body motion capture and force sensors, and include road running instead of treadmill running to discover if changing strike pattern is directly correlated with lower limb injuries, which can be important for training and prehabilitation purposes.

## Figures and Tables

**Table 1:** Three-dimensional contingency table (Shoe*Speed*Foot Strikes).

			Foot Strike Type			
Shoe			Rearfoot	Midfoot	Forefoot	Total
Shod	Speed	Initial	95.0%	5.0%	0%	100%
		Slow	95.0%	5.0%	0%	100%
		Fast	95.0%	5.0%	0%	100%
		Ultimate	90.0%	10.0%	0%	100%
	Total		93.8%	6.3%	0%	100%
Barefoot	Speed	Initial	30.0%	40.0%	30.0%	100%
		Slow	20.0%	45.0%	35.0%	100%
		Fast	25.0%	30.0%	45.0%	100%
		Ultimate	20.0%	35.0%	45.0%	100%
	Total		23.8%	37.5%	38.8%	100%

**Table 2:** K-way effect and partial associations.

	Factors	Chi-Square	*p*-Value
Two-way effects	Overall	85.57	<0.01 *
	Shoe*Foot Strike	99.07	<0.01 *
	Speed*Foot Strike	2.26	0.89
Three-way effect	Shoe*Speed*Foot Strike	0.44	0.99

* *p* < 0.05, the examined factor(s) is considered to be significantly associated.
